# Aneuploidy facilitates dysplastic and tumorigenic phenotypes in the *Drosophila* gut

**DOI:** 10.1242/bio.058623

**Published:** 2021-11-03

**Authors:** Rita Brás, Augusta Monteiro, Claudio E. Sunkel, Luís Pedro Resende

**Affiliations:** 1 Instituto de Investigaçaõ e Inovaçaõ em Saúde, Universidade do Porto, 4200-1353 Porto, Portugal; 2 IBMC – Instituto de Biologia Molecular e Celular, Universidade do Porto, 4200-1353 Porto, Portugal; 3 ICBAS – Instituto de Ciências Biomédicas de Abel Salazar, Universidade do Porto, 4050-353 Porto, Portugal

**Keywords:** Cancer, Mitosis, Aneuploidy, Stem cells, Spindle assembly checkpoint

## Abstract

Aneuploidy has been strongly linked to cancer development, and published evidence has suggested that aneuploidy can have an oncogenic or a tumor suppressor role depending on the tissue context. Using the *Drosophila* midgut as a model, we have recently described that adult intestinal stem cells (ISCs), do not activate programmed cell death upon aneuploidy induction, leading to an increase in ISC proliferation rate, and tissue dysplasia. How aneuploidy impacts ISCs in intestinal tumorigenic models remains to be investigated, and it represents a very important biological question to address since data from multiple *in vivo* models suggests that the cellular impact of aneuploidy is highly dependent on the cellular and tissue context. Using manipulation of different genetic pathways such as EGFR, JAK-STAT and Notch that cause dysplastic phenotypes in the *Drosophila* gut, we found that concomitant aneuploidy induction by impairment of the spindle assembly checkpoint (SAC) consistently leads to a more severe progression of intestinal dysplasia or tumorigenesis. This is characterized by an accumulation of progenitor cells, high tissue cell density and higher stem cell proliferation rates, revealing an additive or synergistic effect depending on the misregulated pathway in which aneuploidy was induced. Thus, our data suggests that in the *Drosophila* gut, both dysplasia and tumorigenic phenotypes can be fueled by inducing genomic instability of resident stem cells.

## Abstract (precis)

**Summary:** Dysplastic and tumorigenic phenotypes are worsened by aneuploidy induction in adult stem cells in the *Drosophila* intestinal epithelium.

## INTRODUCTION

Aneuploidy corresponds to the cellular state in which the chromosome number is not a multiple of the haploid set. While during development, aneuploidy is often associated with embryonic lethality (Gug et al., 2019), some autosomal trisomies are viable (Hassold and Jacobs, 1984), and its impact on adult cells is not necessarily detrimental, as aneuploid cells can be found in healthy tissues, such as the human brain (Rehen et al., 2005; Bushman and Chun, 2013) and liver (Duncan et al., 2012). Furthermore, multiple studies have shown that aneuploidy is associated with aging, neurodegeneration and cancer. However, studies on aneuploidy have highlighted that, in order to understand its impact on cell-fate, factors such as the cell-type and tissue-context must be considered. Many studies have reported a detrimental impact of aneuploidy, leading to cellular stress, cell cycle arrest, or apoptosis (Ohashi et al., 2015; Zhu et al., 2018) but, in other circumstances, it has also been shown to lead to overproliferative phenotypes (Torres et al., 2010; Chen et al., 2012). More recently, work on aneuploidy and its relationship with cancer, has shown that the outcome in cell behavior is highly context-dependent (Ben-David and Amon, 2020). Factors such as cell type, type of tumor, and the type/level of aneuploidy have all been suggested to determine the impact of aneuploidy during tumorigenesis (Giam and Rancati, 2015). Given this context-dependent effect, it is particularly relevant to understand the cell-type specific response to aneuploidy. Embryonic stem cells have been proposed to tolerate and survive as aneuploid, contrary to most somatic cells (Mantel et al., 2007; Ben-David et al., 2014). We and others have shown that adult stem cells can present a similar ability to survive and proliferate as aneuploid (Mirkovic et al., 2019; Harper et al., 2010; Resende et al., 2018). This characteristic of adult stem cells has a potentially strong physiological impact, due to their essential role in tissue maintenance.

This resistance of adult stem cells to aneuploidy has important implications in tumors where they can fuel tumor growth and resistance, functioning as reservoirs for genomic alterations that could have either a pro-oncogenic or tumor suppressor role.

The *Drosophila* intestine is an excellent model system to address the impact of aneuploidy on adult stem cells. It has a high degree of homology with mammals (Reiter et al., 2001), it is amenable to genetic manipulations and multiple markers are available for all different cell types composing the midgut, thus facilitating the characterization of epithelial phenotypes. The *Drosophila* intestine is maintained by the action of multipotent progenitor intestinal stem cells (ISCs) (Ohlstein and Spradling, 2006). In addition to ISCs, enteroblasts (EBs) are another undifferentiated cell type in this tissue, while absorptive enterocytes (ECs), and secretory enteroendocrine cells (EEs) constitute the differentiated cell populations found in the intestine (Biteau et al., 2011). We have recently shown that induction of aneuploidy in *Drosophila* adult ISCs results in a dysplastic phenotype, resembling early stages of tumorigenesis. Once aneuploid, ISCs accumulate and increase their proliferation rate, leading to a higher cell density within the tissue (Resende et al., 2018). Differentiated EEs also accumulate in response to aneuploidy induction within ISCs, suggesting that aneuploidy also impacts the differentiation program. This data provided an *in vivo* model of how aneuploidy could lead to tissue pathology when induced in a healthy/homeostatic context. One of the most relevant questions opened with these findings was whether ISC capacity to survive and proliferate as aneuploid, was maintained by ISCs in an epithelial tumorigenic context, and, if so, what could be the impact of particular type of genomic instability in the progression of this phenotype.

Here, we report experiments describing the effect of inducing aneuploidy in different genetic contexts such as misregulation of EGFR, JAK-STAT and Notch. Aneuploidy was induced in these different genetic backgrounds of intestinal dysplasia and tumorigenesis by depleting SAC proteins. We find that induction of aneuploidy consistently led to a more severe dysplastic or tumorigenic phenotype, when compared to the phenotype observed upon misregulation of the developmental pathways or SAC alone. Our results suggest that in the context of a tumorigenic phenotype, induction of aneuploidy can promote tumor growth, and highlight the importance of more studies in the characterization of resident stem cells ploidy status within epithelial tumor, and its impact on tumor progression.

## RESULTS AND DISCUSSION

### Intestinal dysplasia observed upon aneuploidy induction in ISCs/EBs is milder when compared the misregulation of EGFR or JAK-STAT

Our initial goal was to study the dysplastic phenotypes we have previously reported upon aneuploidy induction in homeostatic ISCs compared when compared with the phenotypes observed upon misregulation of different important developmental pathways, such as EGFR or JAK-STAT. To address this, we evaluated the impact of two alternative conditions of aneuploidy in the intestinal epithelia, and compared these phenotypes with the ones obtained in three independent conditions previously associated with dysplasia.

In this study, we used an already established and efficient strategy to induce aneuploid ISCs in *Drosophila*, through the impairment of the SAC (Musacchio and Salmon, 2007). The SAC is crucial for correct mitotic divisions, as it monitors faithful chromosome segregation ensuring proper cell cycle progression through anaphase. Our previous work demonstrated that inducing aneuploidy in ISCs, leads to stem cell accumulation and overproliferation (Resende et al., 2018).

To induce aneuploidy in ISCs/EBs, we resorted to the binary Gal4-UAS system (Caygill and Brand, 2016), to express UAS-RNAi constructs against two alternative SAC genes (UAS-mad2RNAi or UAS-mps1RNAi) in ISCs/EBs, as previously described (Resende et al., 2018). A temperature sensitive repressor of the GAL4 system was used to block the expression of constructs during the development (flies were kept at 18°C until adults). Upon pupal eclosion, flies were shifted to 29°C in order to drive RNAi expression, allowing the induction of SAC impairment in the adult ISCs/EBs.

**Fig. 1.**
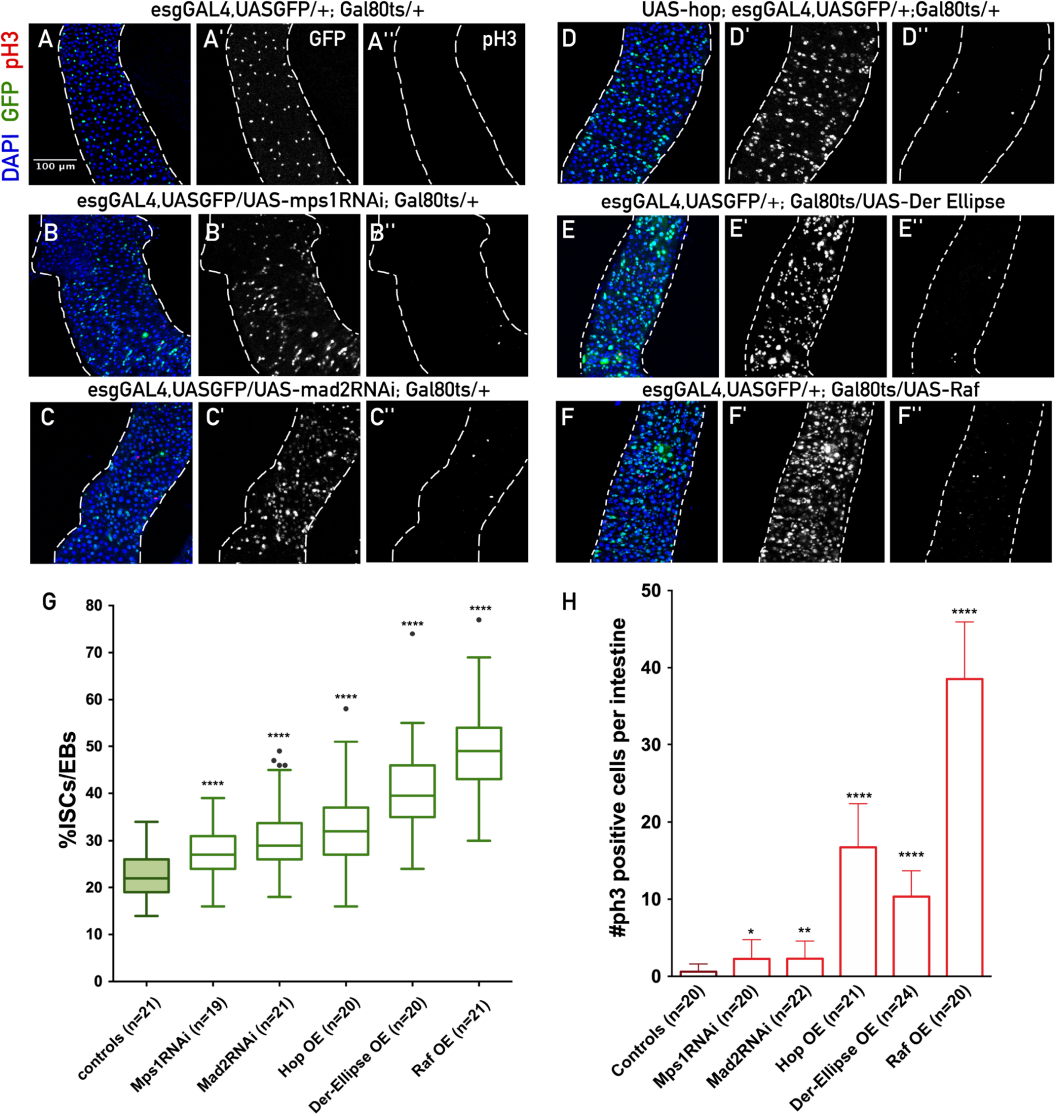
**Aneuploidy induction in ISCs/EBs leads to intestinal dysplasia milder than the one observed upon EGFR or JAK-STAT pathways misregulation.** (A) Control intestines after 10 days at 29°C, ISC/EBs are GFP positive (green, A′, *esgGal4,UASGFP*) and mitotic cells are stained for phospho-histone H3 (red, A′′, no positive cells in this image). (B,C) Intestines where aneuploidy was induced in ISCs/EBs during 10 days at 29°C by expressing UAS-mad2RNAi or UAS-mps1RNAi. (D,F) Intestines where EGFR or JAK-STAT pathways were manipulated in ISCs/EBs during 10 days at 29°C by expressing UAS-hop, UAS-der-Ellipse or UAS-raf. (G) Quantification of the percentage of ISCs/EBs per total cells (DAPI) in A to F; (H) quantification of number of mitotic cells (pH3 positive) in A to F. All images are in the same magnification. *P-value ≤0.05, **P-value ≤0.01, ****P-value ≤0.0001, Mann–Whitney *U*-test.

Intestinal dysplasia in *Drosophila* has been widely characterized by overproliferation of progenitor cells, an impaired differentiation, and changes in epithelial architecture and/or cell shape, being often associated with a pre-malignant state (Apidianakis and Rahme, 2009, 2011). In order to characterize the different phenotypes, we have evaluated epithelium alterations through quantification of the number of ISCs/EBs per total cell number, the number of mitotic cells, and the cellular density within the midgut. In agreement with our published results, when SAC was impaired either through a RNAi-mediated knockdown of *mps1* or *mad2*, we observed a significant accumulation of ISCs/EBs when compared to controls (Fig. 1A–C,G). Accordingly, intestines from flies expressing UAS-mps1RNAi or UAS-mad2RNAi presented a higher number of cells undergoing mitotic divisions (Fig. 1A–C,H), and an increase in epithelial cell density (Fig. S1A–C,M).

The EGFR and JAK-STAT pathways are crucial for stem cell regulation in the *Drosophila* intestine and manipulation of these pathways have been associated with dysplastic or tumorigenic phenotypes. The EGFR/RAS/MAPK is a well-characterized oncogenic pathway with multiple functions in cell behavior, and shown to be implicated in stem cell-derived epithelial cancers, including colorectal cancer (Miyamoto et al., 2017). In the fly midgut, the EGFR pathway plays an important role in the regulation of cell proliferation, growth and epithelial regeneration, and its dysregulation has been associated with tumorigenesis (Biteau and Jasper, 2011; Cordero et al., 2012). Another important pathway for stem cell regulation in the midgut is the JAK-STAT pathway, fundamental for various developmental processes, such as the innate immune response, cellular proliferation, and stem cell development (Rawlings et al., 2004). In the *Drosophila* intestine, this pathway acts as a mitogenic signal important to maintain intestinal homeostasis but also to mediate stress-induced responses (Jiang et al., 2009). A sustained activation of the JAK-STAT pathway is considered as a causal event of tumorigenesis in both *Drosophila* and humans (Amoyel et al., 2014; Trivedi and Starz-Gaiano, 2018).

To address the impact of the misregulation of these pathways in adult ISCs/EBs, we used the Gal4-UAS system, as described previously. Previous studies in *Drosophila* have reported an accumulation of ISCs, upon expression of UAS-raf or UAS-der-Ellipse (EGFR over-activation) (Patel and Edgar, 2014; Ma et al., 2016), or UAS-hop (JAK-STATover-activation) (Markstein et al., 2014; Ren et al., 2015) in ISCs/EBs. We have confirmed these findings and observed that the expression of UAS-hop, UAS-raf or UAS-der-Ellipse in ISCs/EBs, all resulted in an increase in the accumulation of progenitor cells (Fig. 1D–G), higher levels of mitotic ISCs (Fig. 1D–F,H) and higher cellular density (Fig. S1D,G,J,M–P). Interestingly, the severity of the dysplasia observed was stronger in the situations where EGFR or JAK-STAT components were misregulated when compared to the aneuploidy conditions. One possible explanation for this observation could be linked to the fact that ISC/EB proliferation rate is relatively low under homeostatic conditions and mitotic divisions must occur for aneuploid ISCs/EBs to be generated upon SAC impairment. Furthermore, it is expected that only a fraction of those divisions results in mitotic errors that lead to aneuploid ISCs/EBs. Thus, it is expected that for the generation of a significant proportion of aneuploid ISCs/EBs in the midgut, several days are needed, and this is an important factor to be considered when comparing the severity of the dysplastic phenotypes in aneuploid conditions versus manipulation of JAK-STAT or EGFR.

### Aneuploidy induction potentiates dysplasia observed upon EGFR and JAK-STAT misregulation

**Fig. 2.**
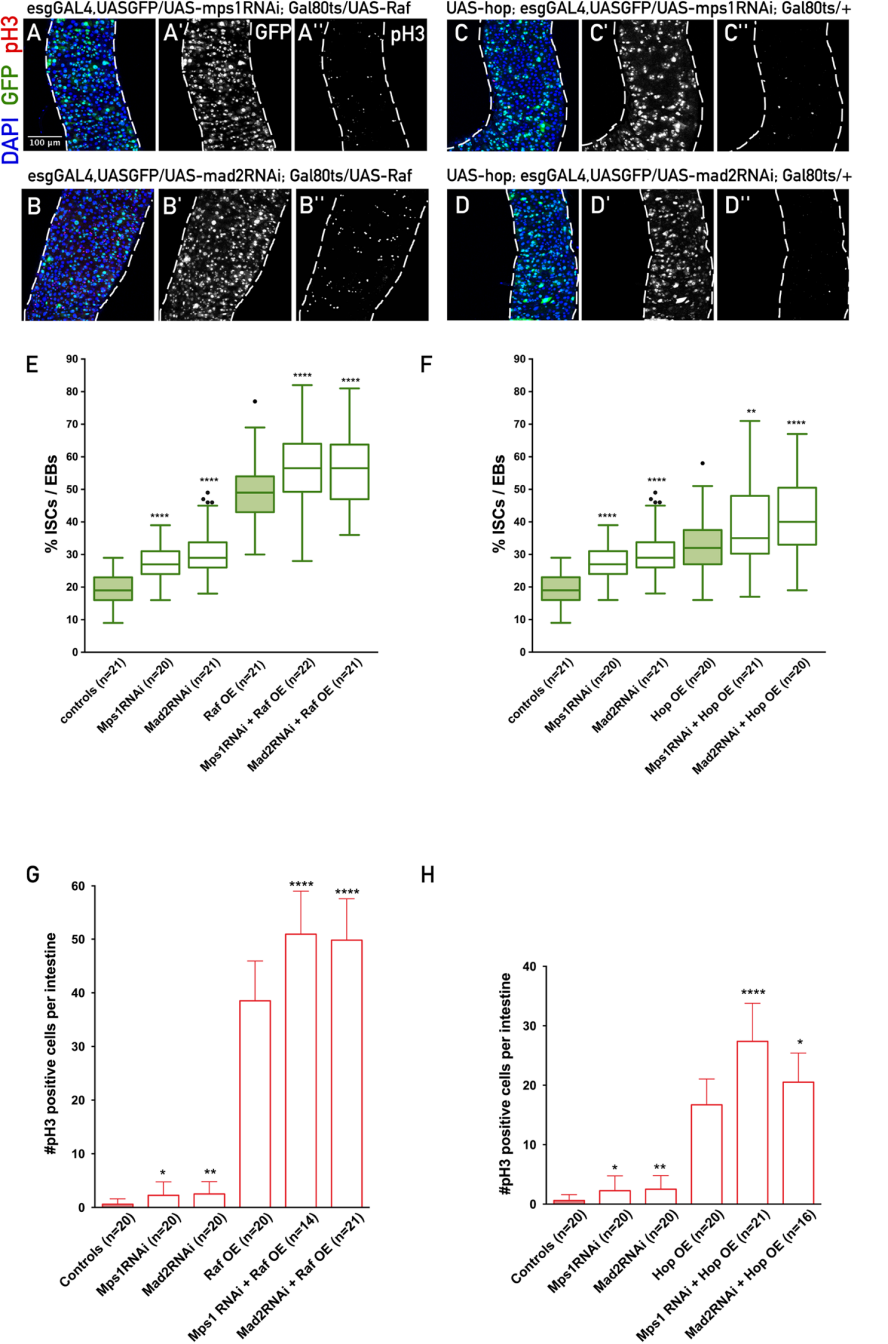
**Aneuploidy induction in ISCs/EBs potentiates the development of EGFR and JAK-STAT dysplastic phenotypes in the midgut*.*** (A,B) Intestines where EGFR pathway was misregulated in ISCs/EBs through expression of UAS-raf, during 10 days at 29°C, with a simultaneous induction of aneuploidy either by expressing UAS-mad2RNAi or UAS-mps1RNAi. (B,C) Intestines where JAK-STAT pathway was misregulated in ISCs/EBs through expression of UAS-hop, during 10 days at 29°C, with a simultaneous induction of aneuploidy either by expressing UAS-mad2RNAi or UAS-mps1RNAi. (E,F) Quantification of the percentage of ISCs/EBs per total cells (DAPI) in controls and A to D (data from controls, Mps1RNAi and Mad2RNAi is the same as in Fig. 1). (G,H) Quantification of number of mitotic cells (pH3 positive) in controls and A to D (data from controls, Mps1RNAi and Mad2RNAi is the same as in Fig. 1). All images are in the same magnification. For statistical analysis, Mps1RNAi and Mad2RNAi were compared with controls, and Mps1RNAi+Raf OE and Mad2RNAi+Raf OE were compared with Raf OE; *P-value ≤0.05, **P-value ≤0.01, ****P-value ≤0.0001, Mann–Whitney *U*-test.

Previous studies have shown that the impact of aneuploidy in cell fate is highly complex, and that it depends greatly on the cellular and tissue context (Giam and Rancati, 2015). Therefore, an important question to address is what would be the impact on midgut epithelial phenotypes of inducing aneuploidy on a context of dysplasia. In order to investigate this, we co-expressed two constructs in ISCs/EBs: one construct to either over-activate the EGFR (UAS-raf or UAS-der-Ellipse) or over-activate the JAK-STAT (UAS-hop), to induce dysplasia, and another one to impair the SAC and induce aneuploidy (either UAS-mps1RNAi or UAS-mad2RNAi). This strategy allowed us to study six different combinations of genetic conditions where dysplasia was induced in combination with or without aneuploidy induction. Regarding EGFR misregulation via RAF over-expression, we observed that aneuploidy induction led to an increase in the number of ISCs/EBs, number of mitotic cells, and cell density when compared to the overexpression of Raf alone (Fig. 2A,B,E,G). Consistently, induction of aneuploidy on the context of EGFR misregulation via an alternative gene (UAS-Der-Ellipse) also resulted in a more severe dysplasia than the one observed with UAS-der Ellipse alone (Fig. S2A–D). Thus, we can conclude that aneuploidy exacerbates the development of dysplasia caused by misregulation of the EGFR pathway.

In order to test if the impact of aneuploidy on the development of dysplastic phenotypes, observed on the context of EGFR manipulation, could be observed in other genetic contexts, we studied this on the context of dysplasia caused by JAK-STAT misregulation. For this purpose, we expressed UAS-hop in ISCs/EBs (JAK-STAT overactivation) while simultaneously inducing aneuploidy with UAS-mps1RNAi or UAS-mad2RNAi. Flies where UAS-hop was simultaneously expressed with UAS-mps1RNAi or UAS-mad2RNAi showed a higher percentage of accumulation of ISCs/EBs, higher number of mitotic cells, and higher tissue cell density (Fig. 2C,D,F,H; Fig. S1J–L,P). Thus, aneuploidy induction led to an increase of the dysplasia observed with the UAS-hop alone. While clearly leading to a more severe epithelial phenotype, induction of aneuploidy acted mostly as an additive effect, when dysplasia was induced by misregulation of either the EGFR and JAK-STAT pathways. Accordingly, we can conclude that aneuploidy induction, in the context of misregulation of EGFR or JAK-STAT promotes the development of dysplasia.

### Aneuploidy induction in ISCs/EBs potentiates the development of the tumorigenic phenotype caused by Notch loss-of-function

**Fig. 3.**
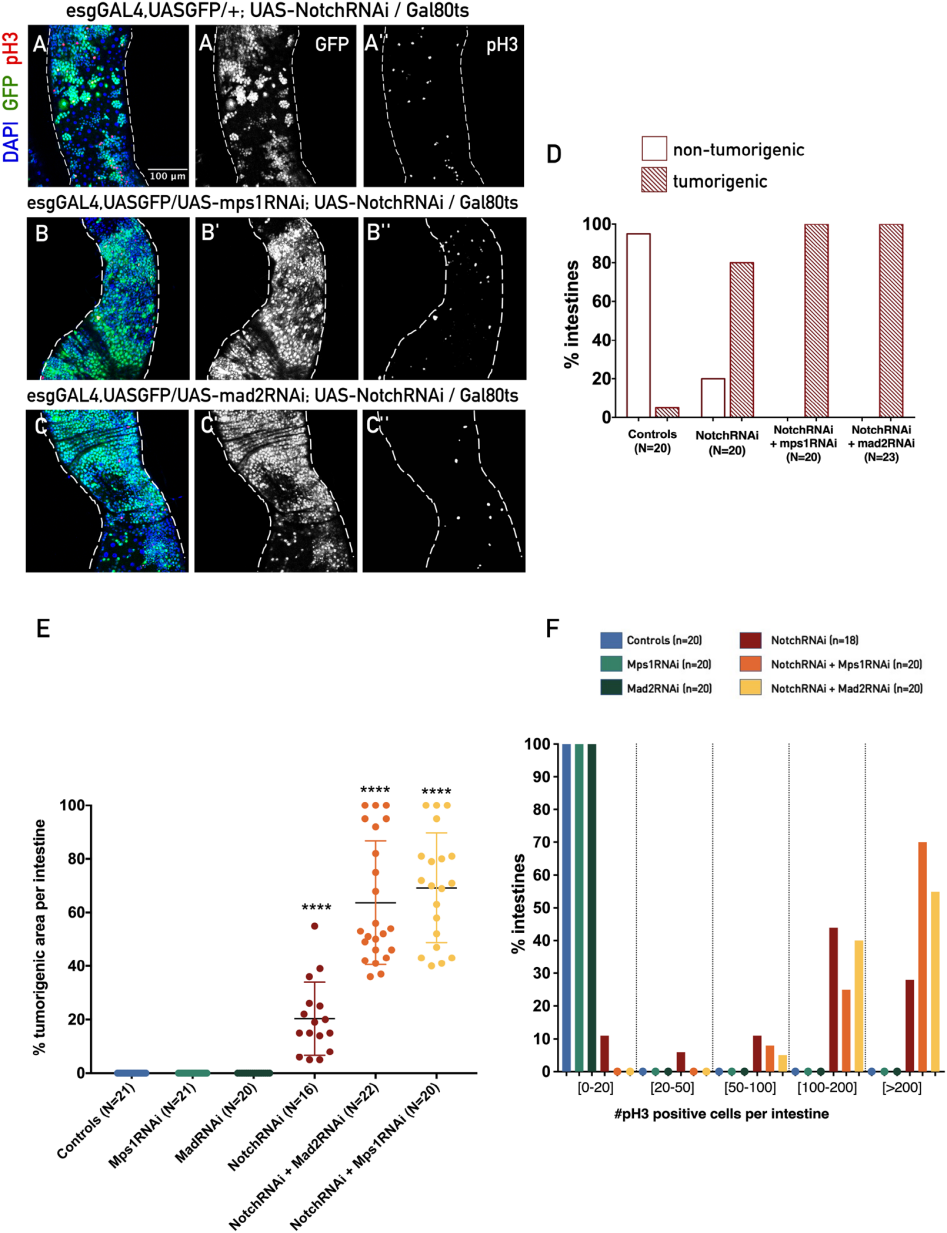
**Aneuploidy induction in ISCs/EBs potentiates the development of tumorigenic phenotypes upon Notch downregulation*.*** (A**)** Intestine where Notch pathway was downregulated in ISCs/EBs through expression of UAS-notchRNAi, during 10 days at 29°C. (B,C) Intestines where Notch pathway was downregulated in ISCs/EBs through expression of UAS-notchRNAi, during 10 days at 29°C, with a simultaneous induction of aneuploidy either by expressing UAS-mad2RNAi or UAS-mps1RNAi. (D) Percentage of intestines presenting a tumorigenic appearance in controls (for image see Fig. 1) and conditions described in A to C. (E) Percentage of tumorigenic area (ISCs/EBs clusters) per total area of the midgut analyzed in controls (for image see Fig. 1) and A to C. Note that for Mad2RNAi and Mps1RNAi conditions although a high number of ISCs/EBs were observed, areas of large clusters of these cells were not found, so the percentage of tumorigenic area was considered zero for both conditions. (F) Percentage of intestines with 0–20, 20–50, 50–100 or >200 mitotic cells (pH3 positive) in controls (for image see Fig. 1A) and in A to C. All images are in the same magnification; For statistical analysis, NotchRNAi was compared with controls, and NotchRNAi+Mps1RNAi and NotchRNAi+Mad2RNAi were compared with NotchRNAi; ****P-value ≤0.0001, Mann–Whitney *U*-test.

The dysplasia phenotypes observed upon EGFR or JAK-STAT manipulation are often portrayed as models for early stages of tumor development, and these can be distinguished from the phenotype observed in the midgut upon manipulation of Notch, another important developmental pathway. Notch is an evolutionarily conserved cell signaling pathway, essential for stem cell maintenance (Liu et al., 2010) and cell fate determination across different developing tissues and organs (Artavanis-Tsakonas et al., 1999; Siebel and Lendahl, 2017). In the *Drosophila* midgut, the level of Notch activity between ISCs that contain the Delta ligand, and the neighboring EB containing the Notch receptor has a determinant role in stem cell fate (Ohlstein and Spradling, 2007; Perdigoto et al., 2011). Mutations that inhibit differentiation in stem cell lineages have been reported in early steps of cancer development (Huang et al., 2015), and in the fly intestine, it has been reported that suppression of Notch signaling results in tumor initiation (Patel et al., 2015). More particularly, in the *Drosophila* midgut, Notch loss-of-function has been shown to lead to the formation of clusters of ISC-like cells that fail to differentiate and proliferate at a very high rate. While EGFR or Jak/Stat overactivation have been associated with a dysplastic phenotype in the fly midgut, Notch downregulation has been characterized as a neoplastic growth. Therefore, decided to address whether aneuploidy could also have an impact on this model. Firstly, and to test whether we could observe the reported Notch loss-of-function epithelial phenotypes (Patel et al., 2015), we expressed a UAS-notchRNAi construct in ISCs/EBs during the first 10 days of the adult life. After 10 days of expressing UAS-notchRNAi in ISCs/EBs, we observed a very strong epithelial phenotype, characterized by a striking accumulation of GFP positive cells that formed several clusters across the midgut, and a very high number of mitotic cells per intestine (Fig. 3A,D–F). This phenotype could easily be distinguished from the ones observed upon aneuploidy induction or EGFR or JAK-STAT manipulation (Fig. 1A), as clusters of a very high number of ISCs/EBs were found in the midgut epithelium (Fig. 3A). The quantification of ISCs/EBs in this phenotype proved to be impossible due to the large number of GFP positive cells, therefore we opted to quantify the severity of the tumorigenic phenotype by the number of intestines that presented a clear tumorigenic appearance (several clusters with large numbers of ISCs/EBs), the percentage of these areas per total area of the midgut, and the number of mitotic cells per midgut. After NotchRNAi expression, we found that 80% of the intestines analyzed presented the phenotype previously described in the literature, with ISCs-like clusters across the midgut (Fig. 3A,D). On average, 20% of the total area of the midgut was occupied by these clusters (Fig. 3E). Moreover, the majority of the Notch depleted intestines analyzed had a number of mitotic cells significantly higher when compared to controls and to the other dysplastic conditions (Figs 1H and 3A,F). We then proceeded to induce Notch downregulation while simultaneously inducing aneuploidy, as describe before for the JAK-STAT and EGFR experiments. Aneuploidy induction in ISCs/EBs had a strong and synergistic impact on the severity of the phenotype caused by Notch downregulation. All intestines had a tumorigenic phenotype (Fig. 3A–D), presented a clear increase in the area occupied by these tumorigenic clusters (Fig. 3A–C,E), and in the number of mitotic cells per intestine (Fig. 3A–C,F). According to a previous study, Notch-defective ISCs require stress-induced divisions for tumor initiation (Patel et al., 2015). Based on these results, we can speculate that aneuploidy might be acting as a source of stress and increase the malignant behavior of ISCs/EBs. Importantly, we could conclude that aneuploidy also exacerbated the development of a neoplastic phenotype. In our experiments, we observed that in EGFR, JAK-STAT tumor models it revealed to have an additive effect, while it had a clear synergistic effect in the case of Notch tumors. Since the biology of these tumors is well understood to be different, being the EGFR, JAK-STAT tumor phenotypes characterized as dysplastic, while Notch tumors are characterized as neoplastic, we speculate this might explain the difference between the additive and synergistic effects of aneuploidy. Future studies should focus on what types of aneuploid genotypes (specific unbalanced chromosomes) in particular are responsible for this effect and on how different pathways crosstalk to increase ISC proliferation. One possible mechanism, might involve the JNK pathway as both aneuploidy induction and EGFR activation have been shown to lead to overactivation of this stress pathway and this overactivation was shown to be necessary for ISC proliferation (Resende et al., 2018; Biteau and Jasper, 2011).

In this work, we characterized the impact of inducing aneuploidy in ISCs, under homeostatic conditions and under contexts of misregulation of developmental pathways associated with dysplastic and tumorigenic phenotypes in the gut. We show that aneuploidy induction in ISCs potentiates the development of intestinal dysplasia and tumorigenic phenotypes driven by misregulation of pathways such as JAK-STAT, EGFR and Notch. Aneuploidy is a source of genomic variability, which has been suggested to confer phenotypic advantages allowing a better adaptation of malignant cells to changing environments (Negrini et al., 2010). Consistently, aneuploidy correlates with resistance to antineoplastic treatments (Lee et al., 2011) and metastatic behavior (Bakhoum et al., 2018). However, there is a great complexity of the phenotypes conveyed by aneuploidy, since it is highly dependent on the type of cancer cells, tissue type and on the tumor microenvironment (Hoevenaar et al., 2020; Ben-David and Amon, 2020). Our work strongly suggests that *Drosophila* midgut stem cells might play a key role in the unveiling of this paradox. In the context of the experiments described here, and the impact of aneuploidy under dysplastic/tumorigenic contexts, we have not addressed a putative non-autonomous contribution of aneuploid stem cell progeny on promoting tumor progression. However, we have previously shown that under homeostatic conditions, the impact of aneuploidy in ISC behavior is driven by an autonomous upregulation of JNK (Resende et al., 2018), as this damage sensing pathway was found to be overactivated in ISCs upon aneuploidy induction and preventing this upregulation specifically in ISCs was sufficient to rescue the dysplastic phenotype. This, together with the fact that ISCs are the only dividing cells in the *Drosophila* intestinal epithelium, represents evidence towards an autonomous effect of aneuploidy to increase the severity of the tumor models here addressed, while future experiments could be planned to address a putative contribution of secreted signals from differentiated progeny to ISCs. When comparing and contrasting our findings in *Drosophila* with mammalian models, one factor to consider is the fact that the *Drosophila* genome is distributed in four chromosomes, a reduced number compared to 23 in humans, or 20 in mice. However, this difference does not seem to have a major impact on how aneuploidy impacts cell biology as *Drosophila* was used as a model for seminal discoveries on the impact of aneuploidy in cell physiology (Birchler, 2013; Milán et al., 2014), and also on the link between aneuploidy and tumor development. Further studies should focus on the characterization of the specific response of stem cells to chromosomal imbalances in different tissues and model organisms, contributing to a better understanding of how aneuploidy impacts human pathologies.

## MATERIALS AND METHODS

### *Drosophila* stocks and husbandry

Flies were maintained on standard cornmeal-molasses-agar medium. Only female progeny from experimental crosses were collected. Less than 30 flies per vial were maintained and turned onto fresh food vials every two days. The following fly stocks used were from the Bloomington *Drosophila* Stock Center (BDSC), Vienna *Drosophila* Stock Center (VDRC), or generous gifts from the fly community as indicated: UAS-mad2RNAi #106003, UAS-mps1RNAi #35283 or #36658, UAS-raf#2033, UAS-hop#14437, UAS-der-ellipse #9533 (Bloomington stock center); UAS-mad2RNAi#44430, UAS-notchRNAi#27229 (Vienna *Drosophila* Resource Centre); esgGal4,UASGFP;Gal80ts (gifts from Dr Leanne Jones, UCLA); wild-type flies were Oregon R. A more detailed information about these stocks is found at Flybase (http://flybase.bio.indiana.edu).

### Immunostaining, microscopy and data analysis

The immunofluorescence (IF) protocol was performed on whole-mount intestines directly dissected in 4% PFA and left ON at 4°C for fixation. After this fixation, three 10-min washing procedures with PBST (PBS 0.1% triton) were carried out and then samples were incubated for 1 h with a blocking solution of PBST/BSA (PBS 0.1% triton 1% BSA). An incubation with primary antibodies was followed, ON and at 4°C. Primary antibodies included: mouse anti-armadillo (1:20) and mouse anti-prospero (1:100) (Developmental Studies Hybridoma Bank, developed under the auspices of the National Institute of Child Health and Human Development and maintained by The University of Iowa, Department of Biological Sciences); rabbit anti-phospho-histone H3 (1:2500) (Millipore); rabbit anti-GFP (1:5000) (Molecular Probes). After this ON incubation, three 10-min washes with PBST were performed and intestines were incubated for 2 h with secondary antibodies. Secondary antibodies were diluted 1:500 (Molecular Probes). Finally, other three 10-min washes with PBST were performed and intestines were mounted in Vectashield mounting medium with DAPI (Vector Laboratories).

Microscopy analysis, of fixed and stained tissues was performed using a 1.1 numerical aperture/40x water objective on an inverted laser scanning confocal microscope Leica TCS SP5 II (Leica Microsystems). The software Leica Application Suite (LAS) Advance Fluorescence 2.6.

The first two fields of view of the posterior midgut (after the pyloric ring) were acquired with a 40x water objective, corresponding to the P3-P4 regions (Marianes and Spradling, 2013) or R4-R5 region (Buchon et al., 2013). Images were taken from both top and bottom layers of the intestines. The N mentioned in this work, corresponds to the number of intestines analyzed. For each intestine, corresponding to an individual N, four images on 40x objective were taken and used for quantifications: two images from the first field of view (top and bottom) plus two images from the second field of view (top and bottom). A minimum of 20 intestines were analyzed from at least two biological replicates – progeny from different crosses, and at least ten of those intestines were used for quantifications. All images were analyzed and edited in the LAS 2.6, and ImageJ 1.50i software; illustrative schemes were obtained with Adobe Illustrator CC 2018; statistical analysis and graphical display were performed using the Prism 7 (GraphPad) software.

## Supplementary Material

Supplementary information
