# Supplementary material for: Aneuploidy facilitates dysplastic and tumorigenic phenotypes in the Drosophila gut
Source: Biol Open. 2021 Nov 3;10(11):bio058623. doi: 10.1242/bio.058623 (PMC8576263; doi:10.1242/bio.058623)
Supplement: Supplementary information [file biolopen-10-058623-s1.pdf]

Supplemental Figures

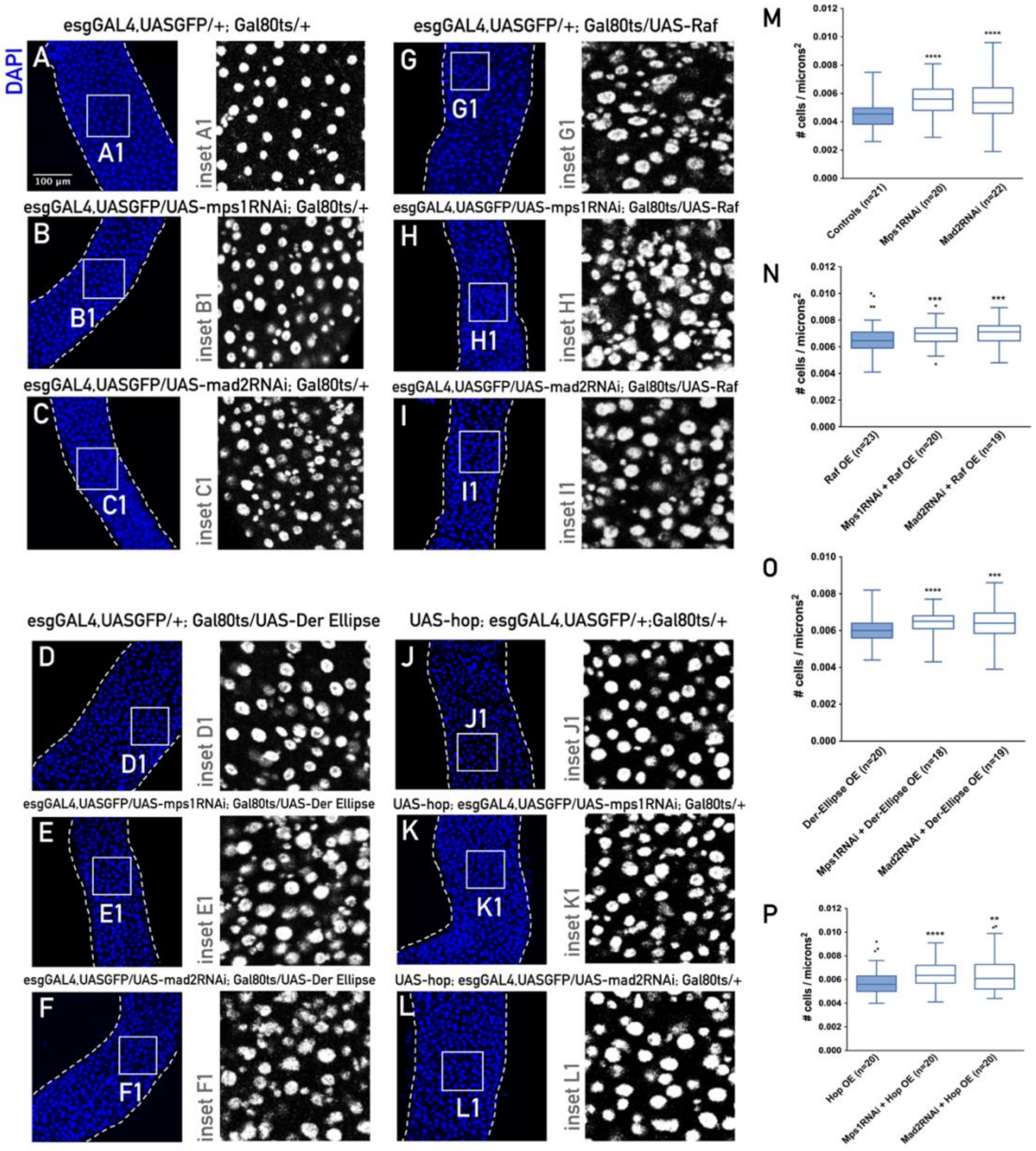

Figure S1. Aneuploidy induction in ISCs/EBs leads to higher tissue cell density when induced in homeostatic intestines and aggravates tissue cell density in intestines developing dysplastic phenotypes upon EGRF or JAK-STAT manipulation. A) Control 10 day-old intestine**; B) and C)** 10 day-old Intestines where aneuploidy was induced via SAC impairment; **D) and L)** 10 day-old Intestines where dysplasia was induced via manipulation of EGFR or JAK-STAT pathways, with and without simultaneous induction of aneuploidy via SAC impairment. **M) to P)** Quantification of numbers of cells (DAPI) per area in situations A) to I); All images are in the same magnification. * p-value ≤ 0.05, ** p-value ≤ 0.01, **** p-value ≤0.001, **** p-value ≤0.0001, Mann–Whitney *U* test.

Biology Open (2021): doi:10.1242/bio.058623: Supplementary information

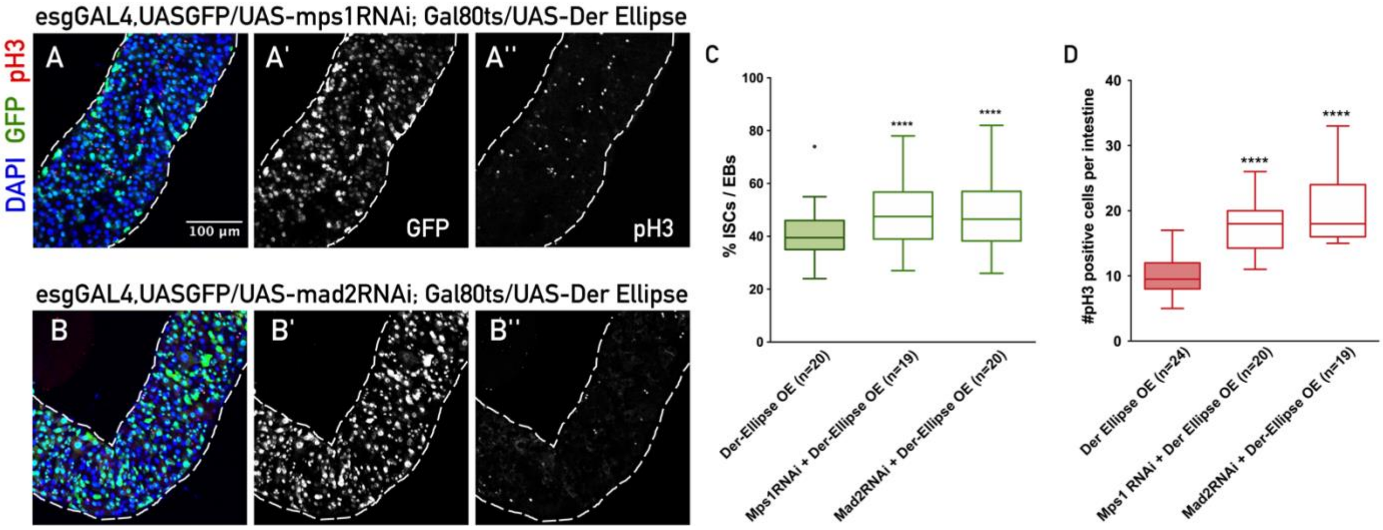

Figure S2. Aneuploidy induction in ISCs/EBs potentiates the development of dysplasia mediated by UAS-der-Ellipse over-expression. A) and B) Intestine where EGFR pathway was activated in ISCs/EBs via expression of UAS-der-Ellipse, during 10 days at 29ºC with a simultaneous induction of aneuploidy either by expressing UAS-mad2RNAi or UAS-mps1RNAi; compared with expression of UAS-der-Ellipse alone and controls in Figure 1. **C)** Quantification of the percentage of ISCs/EBs per total cells (DAPI) in situations A) to B). **D)** Quantification of number of mitotic cells (pH3 positive) in situations A) to F); All images are in the same magnification. * p-value ≤ 0.05, **** p-value ≤0.0001Mann–Whitney *U* test.
